# Reflections on: "The diagnosis of bipolar disorder in pre-pubertal children—what was the controversy about and what did we learn as a result?"

**DOI:** 10.1186/s40345-020-00186-1

**Published:** 2020-04-20

**Authors:** Anthony James

**Affiliations:** Honorary Senior Lecturer, University of Oxford, Oxford, UK

## Abstract

It can be argued that the controversy over paediatric bipolar disorder has been useful in highlighting the issue of bipolar disorder in youths generally. Arising out of this controversy is the recognition that, besides a more uniformed approach to diagnosis, the issue of the disparity in treatments between the US and UK, especially psychopharmacology, needs addressing.

The review of the diagnosis of bipolar disorder in pre-pubertal children (Duffy et al. 2020) was sparked by a very real controversy over an unexpected and dramatic rise in the reported prevalence of prepubertal bipolar disorder (PBD) in the US. One means of exploring scientific disputes such as this, is cross country comparisons. There are two recent examples: (1) the disparity in the diagnosis of schizophrenia between the USA and UK, which resulted in the setting up of WHO epidemiological surveys. These surveys helped clarify the more uniform world-wide incidence of schizophrenia (Leff et al. 1992), (recent surveys have shown a non-uniform incidence (McGrath et al. 2004) (2) The controversy over ADHD alerted to the underdiagnosis in the UK (Prendergast et al. 1998). This led to a profound change in clinical practice in the UK, for the better. Prior to this, children in the UK without a diagnosis of ADHD were disadvantaged, suffering unnecessary and damaging family conflicts, disrupted school careers, and many ending up wrongly in juvenile justice system. In the case of PBD we showed (James et al. 2014) a remarkable–72 fold difference in hospital admission rates between England and the US, which appeared to be due to hospital diagnostic practices, as meta-analyses of epidemiological populations surveys showed a consistency between countries when using narrow, rather than broad, diagnostic criteria (Meter et al. 2019).

While overdiagnosis appears clear in some US studies, a question remains: is there a corresponding under-diagnosis in the UK. One indication might be to look at the transition rates and disparity between adolescent to adult rates of BP between the US and UK (Clacey et al. 2015). While this very indirect measure may give some indication, the question remains unresolved.

There has been the considerable and justifiable criticism of the practice of labeling so many children with PBD, wrongly, so it now clearly appears, with the attendant risk of exposure to unnecessary medication, including antipsychotics and lithium. As these medications have considerable side effects, it became evident that we were in danger of potentially committing an increasing number of children to lifelong, dangerous medications without the evidence to support the diagnosis of PBD in the first place.

Leading on from the question of medication in PBD, there is a largely unnoticed disparity that needs airing about the use of psychopharmacology in the US compared to that in the UK and Europe. The impression is that mediation is used much more frequently in the US (Olfson et al. 2013) at earlier ages, at higher doses, and with more polypharmacy. On the other hand, there is a suspicion that we are under-medicating in the UK and Europe, and an example is lithium, which is rarely used in for adolescents with bipolar disorder in the UK.

Following on from the PBD controversy, a cross country review of medication is needed. However, before that can occur, we need to learn from recent experience. We should resist any blanket criticism of US initiatives, in the belief that private medicine or ‘big pharma’ are in behind any new US approach. That said, the case of Dr Joseph Biederman and his undeclared sponsorship by Johnson & Johnson, manufacturer of risperidone, does highlight a worrisome side of medicine. Furthermore, as one American colleague, pointed out, it would be wise for Europeans to stop treating the US an entity. The controversy over PBD raged as strongly, if not more so, within the US.

So, what do we understand from this? Controversy is good—we can all learn, and our patients benefit. Long live controversy!

## Data Availability

Not applicable.
